# In a secondary care setting, differences between neck pain subgroups classified using the Quebec task force classification system were typically small – a longitudinal study

**DOI:** 10.1186/s12891-015-0609-z

**Published:** 2015-06-16

**Authors:** Hanne Rasmussen, Peter Kent, Per Kjaer, Alice Kongsted

**Affiliations:** The Spine Center of Southern Denmark, Lillebaelt Hospital, Middelfart, Denmark; Department of Sports Science and Clinical Biomechanics, University of Southern Denmark, Odense, Denmark; Nordic Institute of Chiropractic and Clinical Biomechanics, Odense, Denmark

**Keywords:** Neck pain, Classification, Cervical radiculopathy, Quebec task force, Prognosis

## Abstract

**Background:**

The component of the Quebec Task Force Classification System that subgroups patients based on the extent of their radiating pain and neurological signs has been demonstrated to have prognostic implications for patients with low back pain but has not been tested on patients with neck pain (NP).

The main aim of this study was to examine the association between these subgroups, their baseline characteristics and outcome in chronic NP patients referred to an outpatient hospital department.

**Methods:**

This was an observational study of longitudinal data extracted from systematically collected, routine clinical data. Patients were classified into *Local NP only*, *NP + arm pain above the elbow*, *NP + arm pain below the elbow*, and *NP with signs of nerve root involvement* (*NP + NRI)*. Outcome was pain intensity and activity limitation. Associations were tested in longitudinal linear mixed models.

**Results:**

A total of 1,852 people were classified into subgroups (64 % females, mean age 49 years). Follow ups after 3, 6 and 12 months were available for 45 %, 32 % and 40 % of those invited to participate at each time point.

A small improvement in pain was observed over time in all subgroups. There was a significant interaction between subgroups and time, but effect sizes were small. The *local NP* subgroup improved slightly less after 3 months as compared with all other groups, but continued to have the lowest level of pain. After 6 and 12 months, those with *NP + pain above the elbow* had improved the least and patients with *NP + NRI* had experienced the largest improvements in pain intensity. Similar results were obtained for activity limitation.

**Conclusions:**

This study found baseline and outcome differences between neck pain subgroups classified using the Quebec Task Force Classification System. However, differences in outcome were typically small in size and mostly differentiated the *local NP* subgroup from the other subgroups. A caveat to these results is that they were obtained in a cohort of chronic neck pain patients who only displayed small improvements over time and the results may not apply to other cohorts, such as people at earlier stages of their clinical course and in other clinical settings.

## Background

Neck pain is common, with a one-year prevalence of approximately 40 % in the adult population [1], and it contributes substantially to years lived with disability [2]. Neck pain has the potential to cause considerable impact on function and quality of life and thereby, also have large social and financial consequences for individuals as well as society [3, 2, 4].

Clinical definitions and treatment approaches to neck pain vary widely, especially for non-specific neck pain [5, 6]. Even for cervical radiculopathy, a diagnostic sub-category of specific neck pain, there is limited consensus about its diagnostic criteria [7–9]. This lack of diagnostic consensus within the heterogeneity of cervical spine pain disorders, might partially explain why there is such limited knowledge about prognosis and the most effective interventions for neck pain and cervical radiculopathy [10, 3, 11, 12].

The need for identifying subgroups that could improve prediction of outcome and allow better targeting of care has been repeatedly raised as a research priority in spinal pain [13, 14]. The underlying notion is that the heterogeneity of clinical presentations in spinal pain might be better managed and the effect sizes seen in comparative treatment studies might be improved, if valid clinical subgroups were identified that improved treatment selection, afforded greater prognostic precision and better defined appropriate patient selection for clinical trials. In this context, the potential usefulness of subgrouping neck pain was flagged more than 20 years ago when the Quebec Task Force proposed a classification system for spinal pain [15] Since then, a number of other classification systems have also been suggested [16, 17, 6], but none of these have yet matured to the stage of providing strong empirical evidence that they provide better prognostic estimates or treatment outcomes for neck pain patients.

The Quebec Task Force Classification System was proposed as a diagnostic tool for both lumbar and cervical spinal disorders and included 11 categories that are based on pain duration, extent of radiating pain, presence of neurological signs, supplementary examinations and treatment response [15]. The component of this classification that subgroups patients based on the extent of their radiating pain and the presence of neurological signs [15] has been demonstrated to have prognostic implications for patients with low back pain [18–20], but has not been tested on patients with neck pain. It would be helpful to quantify that prognostic value, as currently, there is limited information about the clinical course of patients with cervical pain [21, 22]. Since the Quebec Task Force Classification System is simple and easy to apply in clinical practice, it might have potential for identifying patients at risk of developing chronic pain and disability. Furthermore, the identification of prognostic factors could be a preliminary step to guide research in treatment effect moderators and stratified care [23].

Therefore, the main objective of this study was to examine the association between Quebec Task Force classified subgroups, baseline characteristics and outcome in patients referred to an outpatient hospital department because of their neck pain. Outcome was described by the clinical course of pain intensity, activity limitation and global perceived effect measured at 3, 6 and 12 months after the baseline consultation. A secondary objective was to compare the prognostic capacity of the Quebec Task Force Classification System with that of a more detailed predictive model that included the individual variables that make up the Quebec Task Force Classification system (pain location, pain intensity and number of neurological signs).

## Methods

### Study design

This study was a longitudinal observational study of baseline, 3, 6 and 12-month measurements extracted from systematically collected, routine clinical data.

### Setting

These data were collected at the Medical Department of the Spine Centre of Southern Denmark, which is an outpatient secondary care facility that performs multidisciplinary assessments of patients with back pain referred from either general practitioners or chiropractors in primary care. The main function of the Department is to provide comprehensive patient evaluations via patient history, clinical examination, MRI and other special tests, together with management plans for use by their referring primary care clinician. Short courses of treatment are sometimes offered to clarify treatment response. Data were collected from the Spine Centre’s electronic registry, SpineData. Each patient completed a comprehensive self-reported baseline questionnaire on a touch screen prior to his/her first consultation. The clinician received a summary of the patient’s self-reported data before the clinical examination. Following the clinical examination, the clinician entered the results of a core set of examination procedures into the registry. Patients were also invited to complete two internet-based or postal follow-up questionnaires. Before 1 January 2012, the first follow-up questionnaire occurred 3 months after the date of the initial consultation. After that date, the first follow-up was collected at 6 months, as this was believed to be a more suitable outcome time point in the clinical course of people with chronic pain. The second follow-up questionnaire has always occurred at 12 months.

### Patient cohort

All patients aged 18 years or more referred with neck pain as their main complaint and seen at the Spine Centre between 1 January 2011 and 31 December 2012 were selected for the analysis. This main complaint was self-selected by the patient via a body chart on a touch screen. Patients were excluded if data on pain intensity, or pain location (based on a pain drawing completed as part of the electronic questionnaire), or from the neurological clinical examination were missing.

Data collection in the SpineData database and its analysis in scientific projects has been approved by the Scientific Ethics Committee of the Region of Southern Denmark (Project ID S-200112000-29). Under Danish law, the secondary analysis of such de-identified data does not require separate ethics approval (The Act on Processing of Personal Data, December 2012, Section 5.2; Act on Research Ethics Review of Health Research Projects, October 2013, Section 14.2).

### Definitions of subgroups

Patients were classified into four subgroups adapted from the Quebec Task Force Classification System [15].

#### Local NP only

The pain drawing included only local neck pain and their mean arm pain intensity was zero (mean of the worst pain, the current pain and pain in the preceding 14 days on a 0–10 scale).

#### NP + arm pain above the elbow

The pain drawing indicated pain in the anterior or posterior aspects of the shoulder and/or upper arm, but no pain in the forearm or hand, and mean arm pain intensity was above zero.

#### NP + arm pain below the elbow

The pain drawing indicated pain in the anterior or posterior aspect of the forearm or hand, and mean arm pain intensity was above zero.

#### NP with signs of nerve root involvement (NP + NRI)

The pain drawing indicated any pain in the arm and /or hand, and at least one of the following findings were present on the painful side during clinical examination: muscle weakness, impaired tendon reflexes, altered sensation to touch or pinprick, or a positive Spurling’s test.

### Variables of interest

#### Baseline variables

To describe the four subgroups in the cohort, self-reported variables from the patient questionnaire and clinical examination findings recorded in the clinician’s questionnaire were chosen from the health domains of pain, activity limitation, psychological factors and social factors.

The pain variables included: duration of the current episode of pain (0–3 months, 3–12 months and >12 months), previous neck pain episodes (yes/no), neck pain related to whiplash injury (yes/no) as judged by the clinician based on a clinical interview with the patient, pain irritability (yes to both ‘my pain is easily aggravated by physical activity’ and ‘my pain takes a long time before it settles again’ [24]), neck pain intensity (the 0–10 average of three 0–10 Numerical Rating Scales for each of the following items: ‘current neck pain’, ‘worst neck pain in the last 14 days’, ‘typical neck pain in the last 14 days’ [25]) and arm pain intensity (measured in the same way as neck pain). Dominating arm pain was defined as average arm pain intensity higher than average neck pain intensity.

Activity limitation was measured with a sum score of the Neck Disability Index (0–50 scale, where 0 indicates no activity limitation and 50 indicates severe activity limitation) [26].

Psychological factors were depression, pain-related fear of movement and self-reported general health. Depression was assessed using two PRIME-MD 1000 screening questions [27] (0–10 scales), where patients with a score above 6 on both questions were classified as having depressive symptoms. This cut-point has been validated in this care setting [28] relative to the classification thresholds of the Major Depression Inventory [29]. Pain-related fear of movement was assessed using two screening questions from the Fear Avoidance Belief Questionnaire (0–10 scales). Patients with a sum score ≥14 were classified as expressing fear of movement [30], with the concurrent validity of that cut-point established in this care setting [28], using a threshold (mean plus 1 standard deviation) on the physical activity subscale of the Fear Avoidance Belief Questionnaire derived from the average group scores in five primary care studies. Self-reported general health was assessed using the EuroQOL Health Thermometer (0 = worst imaginable health state and 100 = best imaginable) [31].

Social factors were sick listing, repetitive work, and time spent in static forward head position.

Sick listing was neck pain-related to time off work within the previous 3 months in people in regular employment. Repetitive work was assessed by asking ‘Does your work involve monotonous repetitive movements?’ (0 = ‘not at all’ and 10 = ‘extremely’), and arbitrarily, patients scoring 6 or more were classified as having a high degree of repetitive work. Time spent in a static forward head position was assessed by asking ‘How much of your working time is spent with your neck in forward flexion?’ (0 = ‘no time at all’ and 10 = ‘all the time’), and arbitrarily, a score of 6 or above was used to classify patients as reporting a high degree of working time in neck flexion.

#### Outcome variables

Three, 6 and 12-month outcomes were measured using three variables: pain intensity, activity limitation and global perceived effect. Pain intensity was measured as the higher of either neck pain intensity or arm pain intensity. Activity limitation was measured by the Neck Disability Index. Global perceived effect was assessed using a 7-point Global Perceived Effect Scale [32], for the question ‘How is your neck pain or arm pain now compared with when you started attending the Spine Centre?’ (‘much better, somewhat better, little better, unchanged, little worse, somewhat worse, much worse’). Patients scoring ‘somewhat better’ or ‘much better’ were classified as improved and all other responses as not improved.

No information on diagnosis, treatment prescription or treatment adherence was collected in these data. Therefore, none of those factors were taken into account within this subgrouping, nor in the calculation of outcomes.

### Data analysis

Baseline characteristics were presented as proportions with 95 % confidence intervals (CI), means with standard deviations (SD) or medians with interquartile ranges (IQR). Subgroup differences and outcome responder/non-responder differences were tested using chi-squared, ANOVA or Kruskal-Wallis’ test depending on the type and distribution of the variable.

Associations between the predefined subgroups and outcome measures were tested in longitudinal linear mixed models to account for outcomes having been repeatedly measured over time. Models allowed for random intercept and random slope. The subgroup variable was introduced as a categorical variable in the analysis with dummy variables that had *local NP only* as the reference category. Models with pain and activity limitation were fitted using maximum likelihood estimations and unstructured covariance. The association with the outcome of ‘improved’ global perceived effect was tested in a logistic model. Associations were presented as unadjusted β-values/Odds ratios with 95 % CI. As subgroup differences in episode duration may be a consequence of referral patterns, we first tested if adjustment for duration affected the results, and this was not the case.

The predictive value of the Quebec Task Force subgroups was then compared to the prediction of a more detailed model that consisted of the separate factors of the Quebec Task Force classification: arm pain intensity, dominating arm pain, arm pain location and neurological signs. This comparison was based on linear regression with 12 months of pain and activity limitation outcomes as the dependent variables. To simplify this comparison, it used a single-time outcome rather than a longitudinal model. Variables with associations displaying p-values <0.2 were retained in the model. Predictive capacity was quantified in terms of adjusted R-squared and root mean squared errors (RMSE).

No data imputations were made since only patients with complete baseline variables required for classification into the Quebec Task Force subgroups were included and longitudinal linear mixed models cope with missing outcome values. All analyses were performed using STATA/IC 13.1.

## Results

### Study sample

**Fig. 1:**
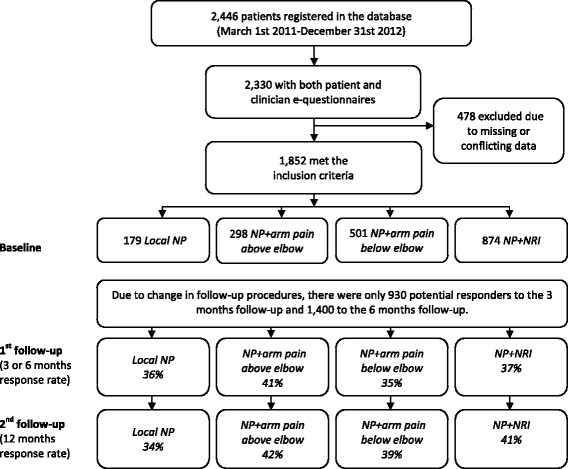
Study patient flow chart

A total of 2,446 patients with neck pain, with or without arm pain, and above 18 years of age were registered within the inclusion period of the study. Of those, 2,330 fulfilled the inclusion criteria, 478 patients were excluded due to missing or conflicting data that were required for the classification process, and therefore 1,852 could be classified into the pre-defined subgroups (64 % females, mean age 49 years) (Fig. 1). As the time period for the initial follow-up changed from 3 to 6 months during the inclusion period, there were only 930 potential responders to the 3-month follow-up and 1,400 to the 6-month follow-up.

Follow ups after 3, 6 and 12 months were completed by 45 %, 32 % and 40 % of those invited to participate at each time point. The proportion of non-responders did not differ across subgroups. There were some statistically significant differences between responders and non-responders. Non-responders were younger (by 3 years at the first follow-up and 5 years at the second), had slightly more activity limitation at baseline (1 and 2 points higher Neck Disability Index scores in non-responders at the first and second follow up respectively), and had a larger proportion of females (62 %) than males (56 %) who did not respond at the 12-month follow-up. Baseline neck pain intensity and arm pain intensity did not differ between responders and non-responders.

### Baseline characteristics

Patient self-reported baseline characteristics are summarised in Table 1 and statistically significant differences across subgroups were observed for many factors. Generally, those with *Local NP* were the least severely affected and those with *NP + NRI* had the most severe profile. These subgroups did not differ significantly on workload factors or sick leave. The differences observed in duration indicated that patients with *NP + NRI* were referred to the outpatient hospital department earlier than other patients, but nonetheless, a large proportion of all subgroups had long-lasting pain, as less than 25 % of patients in all subgroups had an episode duration of 3 months or less and most had a duration of 6 months or more.

**Table 1:** Baseline characteristics of patients in the four neck pain subgroups

	All participants	Local np	Np + arm pain above elbow	Np + arm pain below elbow	Np + nerve root involvement	P-values across subgroups significant pair-wise comparisons
	n = 2,330	n = 179	n = 298	n = 501	n = 874	
Females % (95%CI)	61 (59–63)	65 (58–72)	67 (62–72)	66 (62–70)	60 (57–64)	*p* = .07
Age in years mean (SD)	49 (13)	48 (17)	48 (13)	49 (12)	49 (11)	*P* = .70
Duration,% (95%CI)						*p* < .01
0-3 months	16 (15 – 18)	6 (3 – 11)	10 (7 – 14)	14 (11 – 17)	25 (22 – 28)	local vs. below
3-12 months	36 (34 – 38)	41 (34 – 49)	37 (32 – 43)	36 (32 – 40)	35 (32 – 38)	NRI vs. all
>12 months	48 (46 – 50)	52 (45 – 60)	53 (47 – 59)	50 (46 – 54)	41 (37 – 44)	
Previous episodes	55 (53 – 57)	44 (36 – 51)	58 (52 – 63)	59 (55 – 63)	57 (54 – 61)	*p* < .01
% (95%CI)*						local vs. all
Neck pain intensity median	6 (5 –8)	6 (5–7)	6 (5 – 8)	6 (4 – 8)	6 (5 – 8)	*p* < .04
(IQR)						local vs. NRI
Arm pain intensity	5 (2 – 7)	0 (0 – 0)	5 (2 – 6)	6 (4 – 8)	6 (4 – 8)	*p* < .01 *
median (IQR)						above vs. below
						above vs. NRI
Activity limitation	21 (14 – 27)	16 (12 – 22)	21 (14 – 26)	21 (14 – 27)	22 (15 – 28)	*p* < .01
median (IQR)						local vs. all
Pain irritability	74 (72 – 75)	61 (53 – 68)	71 (66 – 76)	79 (75 – 82)	77 (74 – 79)	*p* < .01
% (95%CI)						local vs. all
						above vs. below
Whiplash % (95%CI)	20 (19 – 22)	22 (16 – 30)	22 (17 – 29)	22 (17 – 27)	17 (14 – 19)	*P* = .07
Depression % (95%CI)	16 (15 – 18)	13 (9 – 19)	18 (14 – 23)	14 (11 – 17)	17 (15 – 20)	*P* = .20
Fear of movement	5 (1 – 7)	3 (1 – 6)	5 (2 – 7)	5 (1 – 7)	5 (2 – 7)	*p* < .01
median (IQR)						local vs. all
General health mean (SD)	50 (24)	56 (23)	51 (23)	48 (25)	48 (24)	*p* < .01 local vs. all
Any sick leave in last 3 months^#^ % (95%CI)	49 (47 – 51)	44 (36 – 53)	44 (38 – 51)	49 (44 – 54)	52 (48 – 56)	*P* = .10
Amount of repetitive work load median (IQR)	3 (0 – 7)	2 (0 – 5)	3 (0 – 7)	4 (1 – 7)	3 (0 – 6)	*P* = .40
Work time with head forward median (IQR)	4 (2 – 6)	4 (2 – 6)	4 (2 – 5)	5 (2 – 7)	4 (2 – 6)	*P* = .05

CI: Confidence intervals; IQR: interquartile range; SD: standard deviation

* test does not include *local NP*

### Associations between subgroups and outcome

**Fig. 2:**
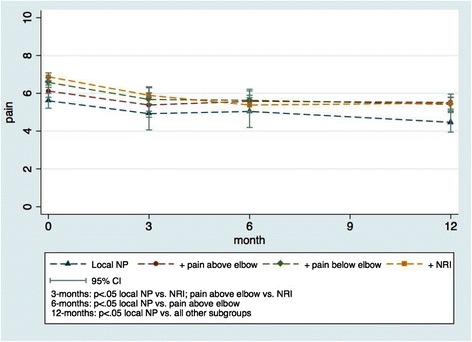
Pain intensity in the four predefined subgroups from baseline to 12-month follow-up

A small improvement in pain was observed over time in all subgroups (Fig. 2). There was an overall statistically significant interaction between subgroups and time, but effect sizes were small (Table 2) and below reported thresholds for clinical importance [25]. The *local NP* subgroup improved slightly less after 3 months as compared with all other groups, but remained with the lowest level of pain. After 6 and 12 months, those with *NP + pain above the elbow* had improved the least (0.6 points improvement after 12 months) and patients with *NP + NRI* had experienced the largest improvement in pain intensity (1.3 points after 12 months) (Table 2).

**Table 2:** Associations between subgroups and outcomes

Outcome	Pain intensity*^,1^	Activity limitation*^,2^
	(0 to 10 scale)	(0 to 100 scale)
	Beta-coefficients	Beta-coefficients
	(95%CI)	(95%CI)
Subgroup effect at baseline		
*Local NP* [reference]		
*NP pain above the elbow*	.5 (−.03 – 1.0)	3.1 (.9 - 5.3)
*NP + pain below the elbow*	1.0 (.5 - 1.5)	3.7 (1.6 - 5.8)
*NP + NRI*	1.3 (.8 - 1.7)	5.5 (3.5 - 7.4)
	*p* < .001	*p* < .001
Time		
3 months	- .6 (−1.2 - .06)	−1.9 (−3.7 - .2)
6 months	- .7 (−1.4 - .06)	−3.4 (−5.1 - -1.1)
12 months	−1.1 (−1.7 - -.5)	−3.3 (−5.2 - -1.4)
	*p* < .001	*p* < .001
Subgroup X time		
3-months		
*Local NP* [reference]		
*NP + pain above the elbow*	-.3 (−1.1 - .5)	-.5 (−3.0 - 1.9)
*NP + pain below the elbow*	-.3 (−1.1 - .4)	-.1 (−2.8 - 2.0)
*NP + NRI*	-.4(−1.0 - .4)	-.8 (−3.2 - 1.2)
	*p* = .8	*p* = .8
6-months		
*Local NP* [reference]		
*NP + pain above the elbow*	.4 (−.4 - 1.3)	.8 (−1.7 - 3.4)
*NP + pain below the elbow*	-.4 (−1.2 - .4)	-.2 (−2.6 – 2.2)
*NP + NRI*	-.8 (−1.5 - .03)	−1.1 (−3.3 - .1.1)
	*p* < .001	p = .2
12-months		
*Local NP* [reference]		
*NP + pain above the elbow*	.5 (−.3 - 1.2)	.4 (−2.0 - 2.7)
*NP + pain below the elbow*	-.1 (−.8 - .6)	.7 (−1.5 - 2.9)
*NP + NRI*	-.2 (−.9 - .4)	-.6 (−2.7 - 1.5)
	*p* = .09	*p* = .3

Longitudinal models used for pain intensity and activity limitation

^**1**^Higher of either average neck pain or average arm pain (n = 1693, average number of observations = 1.6)

^**2**^ N = 1832, average number of observations = 1.7

‘Reference’ indicates the classification category used as the reference in the regression model

**Fig. 3:**
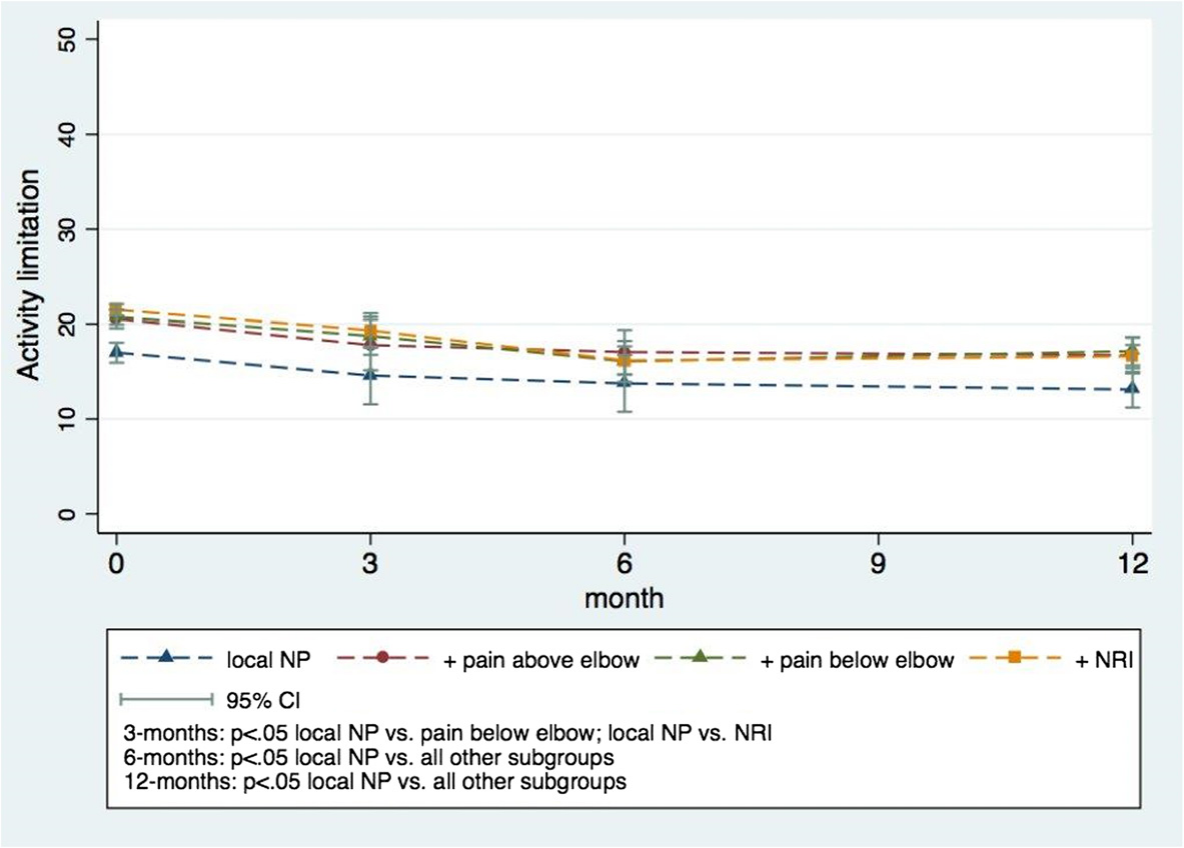
Activity limitation in the four predefined subgroups from baseline to 12-month follow-up

Similar results were obtained for activity limitation (Fig. 3). Improvements were small, and although the overall interaction between subgroups and time was statistically significant, the differences between the trajectories of the subgroups were minor (Table 2) and below reported thresholds for clinical importance [25]. The *local NP* group had the least activity limitation at all the time points, whereas the other groups did not differ significantly from each other at any follow up (Fig. 3).

**Fig. 4:**
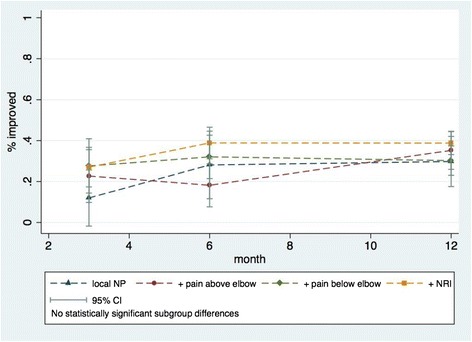
Proportions improved in the four predefined subgroups at three follow-up time points

The proportion of the total cohort reporting improvement (dichotomised global perceived effect) after 3, 6 and 12 months, were 24 % (19 to 30 %), 33 % (28 to 38 %) and 35 % (31 to 39 %) respectively. There was no statistically significant association between subgroups and improvement (Fig. 4.). The estimated odds ratio, as compared with *local NP,* was 1.1 (95%CI 0.6 – 2.1) for *NP + pain above the elbow*; 1.2 (0.7 – 2.2), for *NP + pain below the elbow* and 1.7 (1.0 – 2.8) for *NP+ NRI.* As there was no significant interaction between time and subgroups, these odds ratios apply to all three time points.

### Comparison of the predictive capability of predefined subgroups to that of individual predictors

The model including only *the predefined Quebec Task Force Classification System subgroups* as the predictor variable explained 1.0 % of the variance in the 12-month pain intensity outcome (RMSE 2.41) and 1.4 % in activity limitation (RMSE 10.15). In the *more detailed predictive model*, arm pain intensity, arm pain location, dominating arm pain and having more than one neurological sign were significantly associated with 12-month pain and explained 13 % of the variance in the pain intensity outcome (RMSE 2.26). Arm pain intensity and dominating arm pain were the only significant factors in the more detailed predictive model with activity limitation as the outcome, and this model explained 11 % of the variance (RMSE 9.66).

## Discussion

### Principal findings

This study investigated baseline and outcome differences between neck pain subgroups classified using the Quebec Task Force Classification System. The results showed differences between subgroups at both baseline and in outcome but differences in outcome were small in size and mostly differentiated the *local NP* subgroup from the other subgroups. A secondary objective of this study was to compare the prognostic capacity of these subgroups with that of a more detailed predictive model comprised of variables describing pain location, pain intensity and number of neurological signs. The results showed that the more detailed predictive model had somewhat greater prognostic capacity, explaining between 10.6 % and 13.1 % of the variance in outcome, compared with 1.0 % and 1.4 % for the Quebec Task Force Classification System. These results were obtained in a secondary care cohort of chronic neck pain patients who, on average, only displayed small improvements in pain and activity limitation over time and the results may not apply to other cohorts, such as people at earlier stages in their clinical course.

### Strengths and weakness

The strengths of this study are that the large sample size increased the precision of the estimates, and as these data were collected as part of routine clinical procedures, the results may have generalizability to similar secondary care settings. However, that the spectrum of neck pain patients within sample was predominantly those with more severe and chronic neck pain, might weaken the generalizability of the results. The routine data collection may also weaken the internal validity of the study, as these data may have been of varying quality and more subject to bias than those collected in more rigorous research situations. An additional potential weakness of the study is some uncertainty in the definition of the nerve root involvement, as the reliability and validity of the clinicians’ neurological examinations are unknown and may have resulted in some misclassification of patients. In addition, the Upper Limb Tension Test, recommended to rule out cervical radiculopathy by Rubinstein *et al.* [9], was not part of the standard neurological examination used in the collection of these data. Also, clinicians were not blinded to their patient’s questionnaire when they completed the clinician questionnaire, although this does pragmatically mirror usual practice. We also did not record or control for the type or amount of treatment received over the follow-up period and this could have affected patient outcomes.

It should be recognised that some of the aims of clinical subgrouping are to better target treatment and to stratify care pathways, but information on diagnosis or treatment could be taken into account within the subgrouping or outcome measurement in this study. Hypothetically, if it were possible to systematically and effectively target treatment to the Quebec Task Force classifications, the predictive ability of the subgroups in this study could have been different due to effective treatment differentially improving patient outcomes.

An additional caveat is that the loss to follow up in this study ranged from 58 % to 66 % across the three follow-up points and we have no data quantifying any potential outcome bias between responders and non-responders. In an unpublished study in this same clinical setting, data were available from 200 consecutive *LBP patients* who had not completed the 12-month follow-up questionnaire but subsequently did so when contacted by a research assistant, and their responses were compared with those of 300 randomly selected patients who had completed the questionnaire in the same time period. Non-compliers showed no difference to responders on a wide range of baseline and outcome measures, with the only differences being that non-compliers had (i) more pain intensity at 12 months (5.2 (SD2.6) compared with 4.5 (SD2.7) on a 0–10 scale, p = 0.004), and (ii) had less change in pain intensity from baseline to 12 months (0.8 (SD2.5) compared with 1.3 (SD2.7) on a 0–10 scale, p = 0.048). Although the administrative procedures in this setting are identical for both neck pain and low back pain patients, it is unknown whether this degree of outcome bias would be similar in these neck pain patients.

### Meanings and implications

At baseline, those with *Local NP* were generally the least severely affected and those with *NP + NRI* the most affected. This reflects the findings of another cross-sectional study that found combined neck and arm pain was associated with a lower overall health status compared with either local neck pain or radicular symptoms alone [33]. It is also similar to the findings of a low back pain study that described cross-sectional differences in patients subgrouped using the Quebec Task Force Classification System [34].

On the outcomes of pain and activity limitation, very little improvement was observed over time in all subgroups with small and mostly non-significant differences between groups, although the *Local NP* subgroup had slightly less severe pain at all time points. Similarly, for the outcome of global perceived effect, there were no significant differences between the subgroups at any time point, although the *NP + NRI* group did have slightly higher odds for improvement that was borderline significant. The above findings are in contrast to a recent study of low back pain in the same setting, where the presence of neurological signs and leg pain had prognostic implications for pain and activity limitation, but not for global perceived effect [18] . It could be that the presence of neurological signs actually is less important for prognosis in patients with neck pain, or an alternative explanation might be that in this particular sample of neck pain patients, the neurological examination or recording of its results were less precise than in the low back pain study.

There was a trend towards people in the *NP + NRI* subgroup reporting a higher self-rated improvement despite minimal improvement in pain and activity limitation. It is possible that this reflects patient self-rated global improvement being based on the improvement of symptoms broader than pain intensity and activity limitation, such as those specific to nerve root involvement (improvement in muscle strength, normalisation of sensation and changed quality of pain). This notion might be supported by the more predictive model that points to factors that are related to nerve root involvement as being more associated with a decrease in pain.

The results in this study indicate that simple subgrouping based on the Quebec Task Force Classification System explained very little about the prognosis of individual patients in this chronic neck pain cohort. This suggests that if prognostic models for this setting are to be created that have precision at an individual patient level, they are likely to require more and different factors. It also may be that precision in prognostic estimates at an individual patient level requires predictor variables to remain in their original metrics, as ‘dumbing them down’ into a simple four-category classification loses too much information, no matter how useful the classification might be for other purposes.

Hayden *et al.* [35] suggested that prognostic research should occur within a stepwise framework where identifying and testing prognostic factors is an important initial step towards subsequently understanding prognostic pathways. So, an additional contribution from our results is the indication that individual variables such as pain location, arm pain intensity and number of neurological signs should be considered as candidate variables for prognostic models of neck pain. Although the individual variables that make up the Quebec Task Force Classification System had greater prognostic strength than the Quebec Task Force subgroups, it still did not have sufficient predictive accuracy for us to consider it clinically useful in this setting. However, the Quebec Task Force subgrouping may still usefully reinforce diagnostic categories. Again, a caveat is that this Quebec Task Force Classification may have more prognostic value in less severe and less persistent neck pain populations, especially where treatment is subgroup-targeted. A recent study found that baseline characteristics in patients with nerve-related neck and arm pain predict the likely response to neural tissue management [36], and identifying and matching prognostic factors with relevant treatment pathways has been found to be effective in a large trial of patients with low back pain [37].

The identification of clinically important and validated subgroups of neck pain patients remains a challenge. There is a lack of consistent evidence for prognostic factors, other than baseline pain intensity and baseline disability [38]. To our best knowledge the only externally validated rule for prediction of neck pain outcomes is a rule suggested by Shellingerhout and colleagues which performed well in an external validation sample of chronic neck pain patients [39]. While that model in that rule did include radiating arm pain, it also included other factors that are not contained in the Quebec Task Force Classification. Our finding that the simple Quebec Task Force Classification was not useful for prediction of outcome in a very severely affected group of neck pain patients, suggests a need to also test this more comprehensive Shellingerhout model in populations similar to ours.

## Conclusion

This study found baseline and outcome differences between neck pain subgroups classified using the Quebec Task Force Classification System. However, differences in outcome were small in size and mostly differentiated the *local NP* subgroup from the other subgroups. It also found that, compared with these Quebec Task Force classified subgroups, a more detailed predictive model with variables describing pain location, pain intensity and number of neurological signs, had somewhat greater prognostic capacity, although that more detailed model still did not have sufficient prognostic accuracy for us to consider it clinically useful in this setting. On average, this chronic neck pain cohort only minimally improved in pain and activity limitation over the 12-month follow-up period. A caveat to these results is that this Quebec Task Force Classification System may have more prognostic value in less severe and less persistent neck pain populations, especially where treatment is subgroup-targeted.

## Consent

Under Danish law, the publication of anonymized clinical data does not require patients' consent. However as an additional ethical consideration, 97.7 % of patients in the SpineData registry provide written informed consent for their data to be used for quality assurance and research purposes, including in publications of group-level data.
